# A young woman with traumatic vertebral artery occlusion: a case report 

**DOI:** 10.15171/jcvtr.2018.29

**Published:** 2018-05-21

**Authors:** Amir Ghaffarzad, Reza Javadrashid, Elyar Sadeghi-Hokmabadi, Reza Jamal Arvanaghi, Hassan Soleimanpour, Samad EJ Golzari

**Affiliations:** ^1^Emergency Medicine Research Team, Tabriz University of Medical Sciences, Tabriz, Iran; ^2^Department of Radiology, Imam Reza Teaching Center, Tabriz University of Medical Sciences, Tabriz, Iran; ^3^Neurosciences Research Center, Tabriz University of Medical Sciences, Tabriz, Iran; ^4^Students’ Research Committee, Tabriz University of Medical Sciences, Tabriz, Iran; ^5^Aging Research Institute, Tabriz University of Medical Sciences, Tabriz, Iran; ^6^Research Center for Evidence Based Medicine, Tabriz University of Medical Sciences, Tabriz, Iran; ^7^Road Traffic Injury Research Center, Health Management and Safety Promotion Research Institute, Tabriz University of Medical Sciences, Tabriz, Iran

**Keywords:** Vertebral Artery, Emergency Department, Trauma

## Abstract

Vertebral artery occlusion (VAO) may result from closed head or neck trauma and can be lifethreatening due to brain-stem and cerebellar infarction. CT angiography is recommended as a screening diagnostic tool in selected patients after blunt cervical trauma. A 24-year-old woman was admitted to our emergency department with left hemiplegia two days after motor vehicle collision. Final diagnosis of occlusion of the right vertebral artery was made in CT angiography. She was treated with anticoagulant for 4 days then discharged with 5/5 muscle forces. She was advised to continue warfarin and atorvastatin for her after discharge.

## Introduction

Vertebral artery occlusion (VAO) was first described by Riechert in 1952 in angiography of a patient with a brain-stem syndrome; later, similar cases were reported.^1^ VAO is a potentially life-threatening scenario and may cause serious and even fatal neurological deficits due to brain-stem and cerebellar infarctions. This may result in stroke or may give rise to isolated or combined symptoms and signs of altered consciousness, speech defects, diplopia, blurred vision, nystagmus and dysphagia.^2^ Bow Hunter’s syndrome is a mechanical occlusion of the VA due to physiological head rotation.^3^ Vertebral artery injury (VAI), may result from penetrating injuries, chiropractic manipulation, prolonged abnormal positioning of the neck, birth trauma or from closed head or neck trauma. Nevertheless, damage to the artery as a result of closed injury is considered to be rare.^2^

### Diagnostic recommendations

In certain patients suffering from blunt cervical trauma and meeting the modified Denver screening criteria for suspected VAI, computed tomographic angiography (CTA) is a highly suggested screening modality. Modified Denver criteria are broadly used screening indices which are effective specially for the early diagnosis of blunt cerebrovascular injury (BCVI). BCVI might be associated with some signs and symptoms on the physical examination; these findings include but not limited to cervical thrill or bruit, seatbelt sign, cervical hematoma, massive epistaxis, lateralized neurologic deficits, anisocoria, and Homer’s syndrome. Further imaging studies might reveal basilar skull, cervical spine and facial fractures. Brain CT scan might also be indicative of infarctions. Nevertheless, no significant CT findings might be found in some patients even with Glasgow Coma Scale scores of 8 or less. We suspected BCVI in our case considering the accompanying focal neurologic deficit and modified Denver screening criteria. Furthermore, patients with BCVI can also be categorized into five subgroups based on Vascular Injury Scale (VIS):

Grade I: luminal narrowing <25% in a dissected artery
Grade II: luminal narrowing ≥25% in a dissected artery
Grade III: vertebral artery pseudoaneurysm
Grade IV: vertebral artery occlusion
Grade V: vertebral artery transection

Accordingly, our patient was in Grade IV VIS subgroup.

VAI can be diagnosed using catheter angiography in certain patients suffering from blunt cervical trauma. It is often used when either simultaneous endovascular therapy is considered or CTA is unavailable. Blunt cervical trauma induced VAI might be diagnosed using Magnetic resonance imaging (MRI), especially in those with either complete spinal cord or vertebral subluxation injuries.^4^

## Case Report

**Figure 1:**
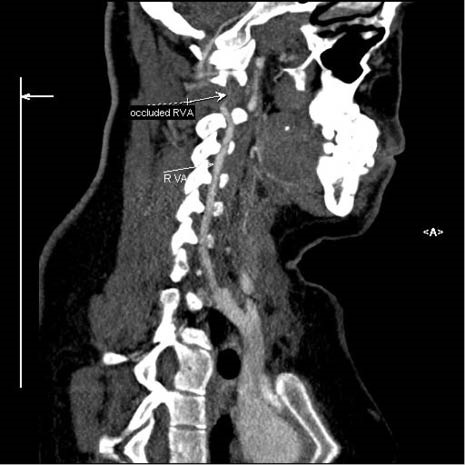
Sagittal curved planar reformatted image or RVA shows cut off at v2 segment.

**Figure 2:**
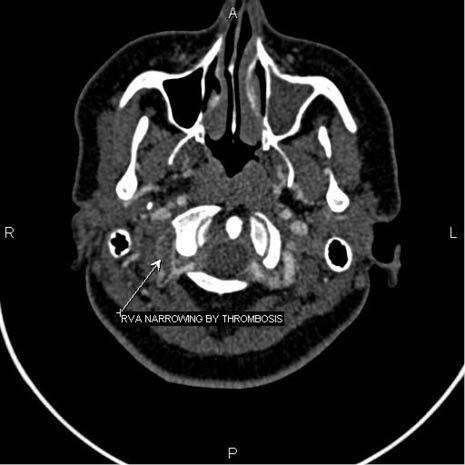
Axial image shows RVA narrowing and irregularity.

**Figure 3:**
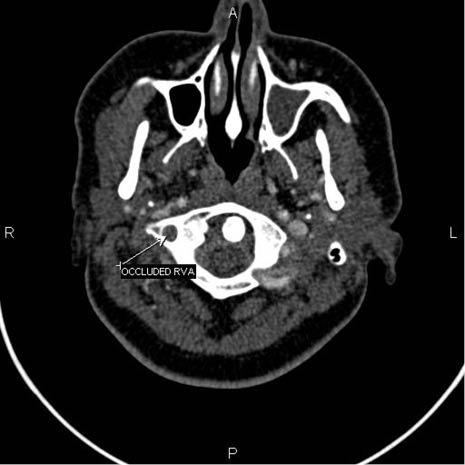
Axial image shows RVA occlusion.

A 24-year-old woman, following motor vehicle collision (MVC), was admitted to the Emergency Medicine Department of Imam Reza hospital, Tabriz, Iran. She was fully oriented with a GCS of 15. Her vital signs were within normal ranges. She complained of mild cervical pain. In neurologic examination, she had no focal neurological deficits. Primary and secondary trauma care was provided. Diagnostic studies including Brain CT scan, cervical and chest radiography and FAST examination were performed which were of unremarkable findings. Consequently, she was discharged after being under observation for 8 hours. Nevertheless, the patient was readmitted to the emergency department with left hemiplegia two days later. Doppler sonography of right vertebral artery reported the probability of dissection or thrombosis; however, CT angiography (Figures 1-3) confirmed the occlusion of the right vertebral artery. Patient was admitted to the neurology ward. Immediately, heparin (1000 unit per hour) was initiated; titration was performed to a PTT target range of 50-70 seconds. Subsequently, warfarin (5 mg per day) was initiated to reach the target INR of 2-3. Additionally, atorvastatin 40 mg was prescribed due to the unknown etiology of the stroke on admission. Cholesterol lowering agents are not considered as part of the standard treatment regimen in patients with large vessel dissection. Nevertheless, we decided to continue atorvastatin as our patient also suffered from dyslipidemia. Physiotherapy of the left extremities was initiated. Her left hemiplegia recovered after four days and she was consequently discharged with 5/5 muscle forces. Warfarin was continued for her after discharge.

## Discussion

VAI following non-penetrating cervical spinal trauma has been reported to be of an incidence of 11%. Modified Denver screening criteria for BCVIs are the most commonly used criteria for the classification of this event.^4^

It is believed that VAI is more frequent following blunt trauma than penetrating trauma which might be as the result of the immediacy of the vertebral artery to bony elements.^5^

Vertebral artery is extremely vulnerable to injuries or occlusions which might occur due to hyperrotation, hyperextension, cervical traction or cervical fracture dislocation. Atlanto-occipital and atlanto-axial joints, and fifth and sixth cervical vertebrae are common sites for combined fracture dislocation and compression. Nevertheless, atlanto-occipital and atlanto-axial joints, and foramen magnum are common sites for arterial stenosis and/or occlusion caused by hyperrotation or hyperextension. Ischemic syndrome of the brain stem or spinal cord are conditions which might rise following bilateral obstruction of vertebral arteries, obstruction of a single vertebral artery combined with inadequate flow in the counterpart artery and finally, embolism, atresia or thrombosis of the posterior communicating or nearby collateral arteries. Rapid excessive rotatory movement or hyperextension of the head with no associated fracture or dislocation are scenarios following which ischemic brain stem or spinal cord syndromes might be seen.^6^

Although there is no documented decisive evidence for the treatment of VAI, those with symptomatic VAI are commonly treated using anticoagulation or antiplatelet agents. Nevertheless, anticoagulation therapy should be used cautiously in multiple trauma patients with VAI due to the dreaded risk of fatal hemorrhagic complications derived from anticoagulation therapy. On the other hand, aspirin, the most widely studied antiplatelet agent, might be considered as a relatively safe regimen in symptomatic patients with VAI following blunt trauma. Currently, therapeutic approach for patients with VAI should be tailored considering numerous elements including but not limited to each patient’s associated traumatic injuries, specific vertebral artery injuries, and also weighing risk/benefit of anticoagulation or antiplatelet agents in every individual patient.^4^

Compared with surgical approaches, vertebral artery stenting (VAS) can be of relatively high success rate yet few intra or post-procedural complications if practiced by experienced interventionists. Symptom might resolve completely or partially in most patients^7^; therefore, VAS should be opted for as a first-line therapy in patients with VAI.^7^

Percutaneous transluminal angioplasty (PTA) is another valuable rescuing procedure that might be considered in those with appropriate vertebral artery.^8^ The important lesson to be learned in this case is the possibility of late presentation of neurologic signs in patients suffering dissection. In traumatic patients, especially those with neck trauma and pain with late neurologic signs, dissection should be considered.

## Acknowledgments

The authors are grateful to all the health personnel and patient who participated in the study.

## Ethical approval

An institutional ethical approval was obtained for this work from Tabriz University of Medical Sciences and a signed written informed consent form was obtained from patient.

## Competing interests

The authors declare no conflict of interests.
